# Error

**Published:** 1898-10

**Authors:** 


ERROR.—The editorial on "The Medical Press," reproduced in our last issue, was from Dr. J. D. Emmet's journal, *The American Gynecological and Obstetrical Journal*, but was improperly credited to "*The American Journal of Gynecology and Obstetrics.*"

